# Ohio State Journal of Dental Science

**Published:** 1881-03

**Authors:** 


### OHIO STATE JOURNAL OF DENTAL SCIENCE.

Since the issue of the last number of the Register the first number of the above-named journal has made its appearance; and its matter and appearance is certainly equal to, if not beyond the sanguine expectation of Dr. Watts' warmest friends. Its matter is throughout attractive, interesting and instructive. It is different from any other dental journal that exists or ever did exist, and on this account it fully justifies the statement that there is room for another journal. The publishers, in its paper and execution have done splendid work, and in appearance it is equal to if not superior to any other dental journal published.

The Register believes in having just as many children in the family (profession) as can be taken care of. We hope that often the Register will help to broadcast the good things of the Journal. Long may it live, and greatly may it prosper.

